# Lifetime Estimation of the Upper Stage of GSAT-14 in Geostationary Transfer Orbit

**DOI:** 10.1155/2014/864953

**Published:** 2014-10-29

**Authors:** Jim Fletcher Jeyakodi David, Ram Krishan Sharma

**Affiliations:** Department of Aerospace Engineering, Karunya University, Karunya Nagar, Coimbatore 641114, India

## Abstract

The combination of atmospheric drag and lunar and solar perturbations in addition to Earth's oblateness influences the orbital lifetime of an upper stage in geostationary transfer orbit (GTO). These high eccentric orbits undergo fluctuations in both perturbations and velocity and are very sensitive to the initial conditions. The main objective of this paper is to predict the reentry time of the upper stage of the Indian geosynchronous satellite launch vehicle, GSLV-D5, which inserted the satellite GSAT-14 into a GTO on January 05, 2014, with mean perigee and apogee altitudes of 170 km and 35975 km. Four intervals of near linear variation of the mean apogee altitude observed were used in predicting the orbital lifetime. For these four intervals, optimal values of the initial osculating eccentricity and ballistic coefficient for matching the mean apogee altitudes were estimated with the response surface methodology using a genetic algorithm. It was found that the orbital lifetime from these four time spans was between 144 and 148 days.

## 1. Introduction 

The estimation of the orbital lifetime of decaying upper stages is important, due to the impact risks associated. In fact, the geostationary transfer orbit (GTO) is a high eccentric orbit which traverses low Earth orbit (LEO) and geostationary orbit (GSO), where the threat of collision with an operational satellite is high. Hence, minimizing the lifetime of an upper stage is a significant consideration in space debris mitigation endeavors. In order to reduce the collision probability, GSO satellites are placed in graveyard orbits at the end of their operational life.

Lunar and solar perturbations on geostationary transfer orbits account for the oscillation of the eccentricity keeping the semimajor axis constant [1]. The effect of lunar-solar perturbations in reducing the orbital lifetime of high eccentricity orbits is also the topic of interest of earlier studies [1–3]. An accurate simulation of the process requires a good estimate of the initial state [4]. Since the semimajor axis is directly related to the period of an orbit, it can be easily measured. Hence, the eccentricity and the ballistic coefficient which depends on the drag coefficient *C*_*D*_, the mass of the object *m*, and the effective area *A* are considered as uncertain parameters [5].

In this paper, a method to perform the lifetime estimation of an upper stage in GTO is presented as an optimal estimation problem [5, 6]. Using this method we predict the reentry time of the cryogenic stage of the Indian geosynchronous satellite launch vehicle GSLV-D5, employing the orbital data of the rocket body (R/B) in the form of two-line element sets (TLEs), which were downloaded from the Space Track Organization website (http://www.spacetrack.org/) from February 11, 2014, to April 6, 2014. Based on near linear variation of the mean apogee altitude, four intervals were identified for the reentry study [6]. The response surface method with genetic algorithm (GA) is effectively used to determine the optimal initial estimates of eccentricity and ballistic coefficient using the TLEs of each time interval [7]. The reentry time estimation in each case is computed using the Numerical Prediction of Orbital Events (NPOE) software by C. David Eagle (http://www.cdeagle.com/html/npoe.html). The influences of lunar-solar perturbations, Earth's oblateness, and atmospheric drag are considered to predict the reentry time more accurately [1, 8, 9].

## 2. Method of Prediction

The orbital elements data given as two-line element sets (TLEs) were downloaded from the Space Track website from February 11, 2014, to April 4, 2014. Since the orbital period was greater than 225 minutes, the SDP4 orbit propagator model [10, 11] was used in the satellite tracking SatSpy software (http://www.satspy.com/) to get the state vectors, consisting of position and velocity, at the particular epochs chosen for the reentry time predictions of the GSLV R/B. SDP4 extends the SGP4 model to lunar-solar gravity perturbations, solar radiation pressure effects, and Earth resonance terms. The state vectors obtained are then given in input to the NPOE software to obtain the mean and osculating orbital elements at the initial state for the orbit simulation. The initial values of the uncertain parameters, ballistic coefficient and eccentricity, are estimated with the response surface technique using a genetic algorithm for the four time intervals where a near linear variation of the mean apogee altitude is observed. Using these initial values for each time interval, the reentry time of the GSLV R/B is predicted. The observed and predicted values of solar flux (*F*_10.7_) and geomagnetic index (*A*_*p*_) data utilized for density computations are obtained from the Solar Terrestrial Activity Report by Jan Alvestad (http://www.solen.info/solar/).

### 2.1. Response Surface Methodology Using Genetic Algorithm

The response surface methodology (RSM) is a combination of mathematical and statistical techniques used widely in various fields for the purpose of development of models and optimization [12]. The objective of using RSM is to optimize a response (output variable) which is influenced by a number of independent variables (input variables) [13, 14]. Consider the response *y* influenced by two independent variables *x*_1_ and *x*_2_, which can be represented mathematically as follows:

(1) $$\begin{array}{c} y=f\left(x_{1},x_{2}\right)+ɛ, \end{array}$$

where *ɛ* is the random error observed in the response *y*. The surface characterized by *f*(*x*_1_, *x*_2_) is called the response surface.

The actual relationship between the response and the independent variables is usually unknown. Therefore, the first step in RSM is to find the approximate model function which shows the true relationship. Generally, the approximation is started with a low-order polynomial function. If the response is characterized by a linear function, then a first-order model is used as approximation function. If the response surface has a curvature, then a higher-order polynomial function is used, such as a second-order model. The objective of RSM is not only to understand the contour of the response surface, but also to estimate the values of independent variables called “design variables,” for which the optimal response is obtained.

In order to obtain the optimal response, a genetic algorithm (GA) which belongs to the group of evolutionary algorithms (EA) is used [15, 16]. In the genetic algorithm there are six steps to be taken.

1. Creation of an initial population: an initial set of solutions or chromosomes is generated randomly or by a seeding procedure.
2. Fitness evaluation: the quality of each solution of the population is evaluated using the fitness function.
3. Selection: with this process promising solutions are chosen to pass to the next generation at the expense of other solutions which are considered ill-equipped for the objective.
4. Crossover: this process consists of taking two strings or parents from the population and performing a random exchange of portions between them to form a new solution. This new chromosome has information from both parents. The crossover does not apply to the entire population string, but it is limited by the crossover rate.
5. Mutation: this involves making changes in individual values of variables in a solution. Mutations serve to maintain the diversity of the population, reducing the probability of finding a local minimum or local maximum rather than the global optimal solution.
6. Checking if the stopping criterion is satisfied: if the stopping criterion is not satisfied, the process returns to step number 3. If the criterion is satisfied, the algorithm finishes.

**Figure 1:**
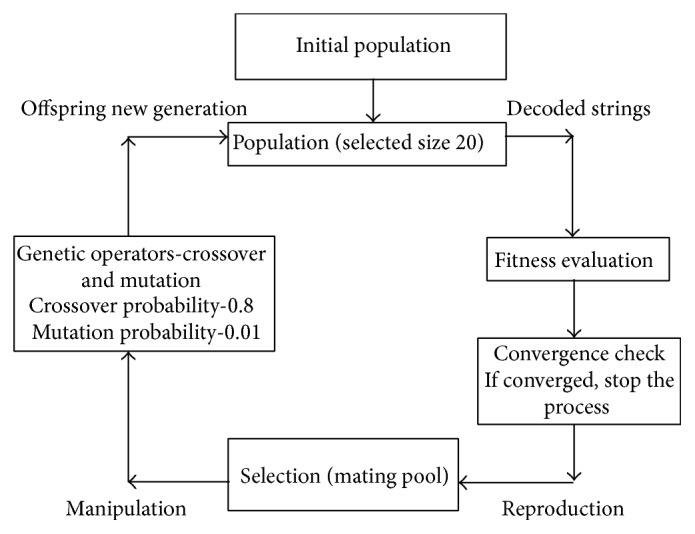
Cycle of genetic algorithm.

**Figure 2:**
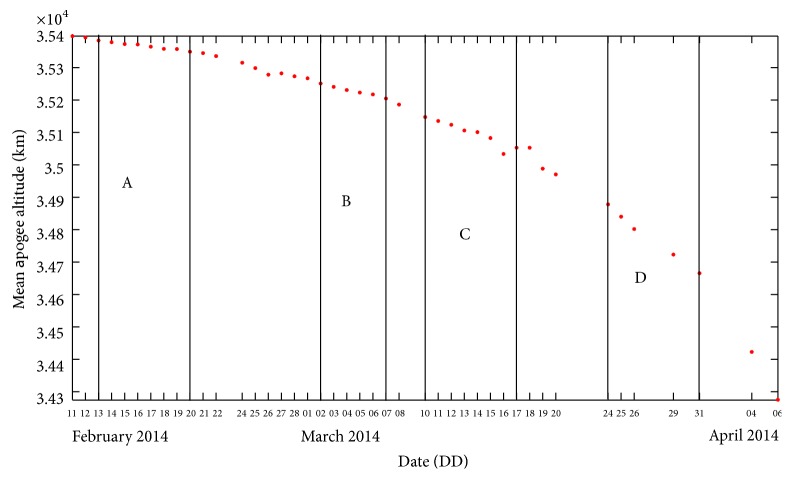
Variation of observed mean apogee altitude of GSLV R/B.

For the reentry time prediction, we consider the initial osculating eccentricity (*e*) and the ballistic coefficient (*B*) as the design variables to find the initial state. The mean apogee altitude generated for the considered initial osculating eccentricity and ballistic coefficient, referred to as mean apogee surface [4], is the response surface. The topography of the mean apogee surface reveals the dynamics of epochal motion. Four time intervals shown in Figure 2 are chosen based on near linear variation of the mean apogee altitude. Each time interval is referred to as a “zone.” Then, we select three initial values of the osculating eccentricity (*e*1, *e*2, *e*3) and the ballistic coefficient (*b*1, *b*2, *b*3) such that the observed mean apogee altitudes fall well within the lower and the upper bounds of the mean apogee surfaces. With the selected initial values of *e* and *B*, we generate nine mean apogee surfaces. Each surface is generated by propagating the trajectory with the selected initial values of *e* and *B* using NPOE. These mean apogee surfaces are used to compute the predicted mean apogee altitude at a specified epoch by interpolation [17]. The genetic algorithm is used to find the optimal solution of the initial values of osculating eccentricity and ballistic coefficient which match the mean apogee altitude. The population size is considered to be 20. Because all optimization parameters have a specified range, a binary coded GA is utilized and all parameters are coded in 60 bits. A single point crossover probability of 0.8 and mutation probability of 0.01 are selected. The cycle of the genetic algorithm is given in Figure 1.

## 3. Reentry Time Prediction for GSLV-D5

**Figure 3:**
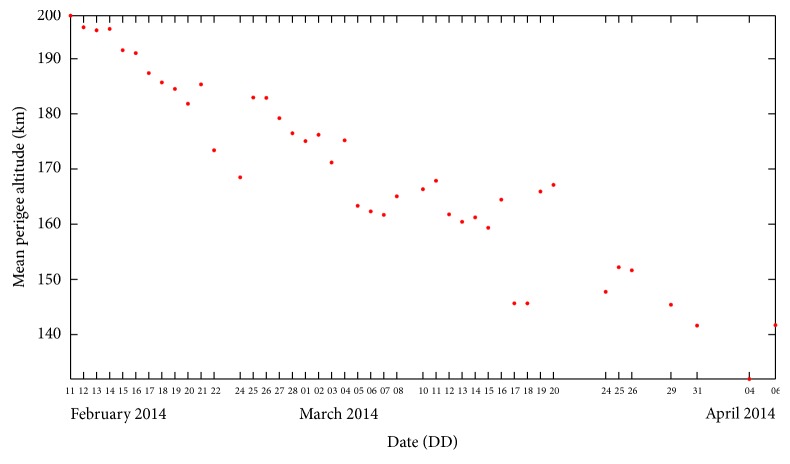
Variation of observed mean perigee altitude of GSLV R/B.

The TLEs downloaded from the Space Track Organization website are converted into state vectors using the SatSpy software and the mean and osculating orbital elements are computed using the NPOE software. The observed mean apogee and perigee altitudes computed from the TLEs are plotted in Figures 2 and 3, respectively. The identified four zones between February 11, 2014, and April 6, 2014, are labelled as A, B, C, and D, respectively. Then, the initial osculating eccentricity and ballistic coefficient are computed for each of the considered zones using the above methodology. The optimal values of *e* and *B* are used in the NPOE software to predict the reentry time, keeping the other orbital elements unchanged. For the orbit propagation using the NPOE software, the terms up to *J*_10,10_ of the Earth gravity model based on GEM10B [18] are considered. For the atmospheric drag perturbations, the MSIS90 density model [19], which includes the observed and predicted values of solar flux and geomagnetic index, is used. The lunar and solar perturbation forces were also included. The mean perigee altitude has gone below 140 km in Figure 2 due to the solar perturbations which has dominated the forces acting on the object.

### 3.1. Case Studies

#### 3.1.1. Zone A

The osculating orbital elements of GSLV R/B (NORAD No. 39499) as obtained from the TLE on February 13, 2014, 15 : 24 (UTC), are as follows:

 semimajor axis (km) = 24163.152291,
 eccentricity = 0.7279715192,
 inclination (°) = 19.33662306,
 argument of perigee (°) = 207.14804219,
 right ascension of the ascending node (°) = 196.09051738,
 true anomaly (°) = 152.63951389.

**Figure 4:**
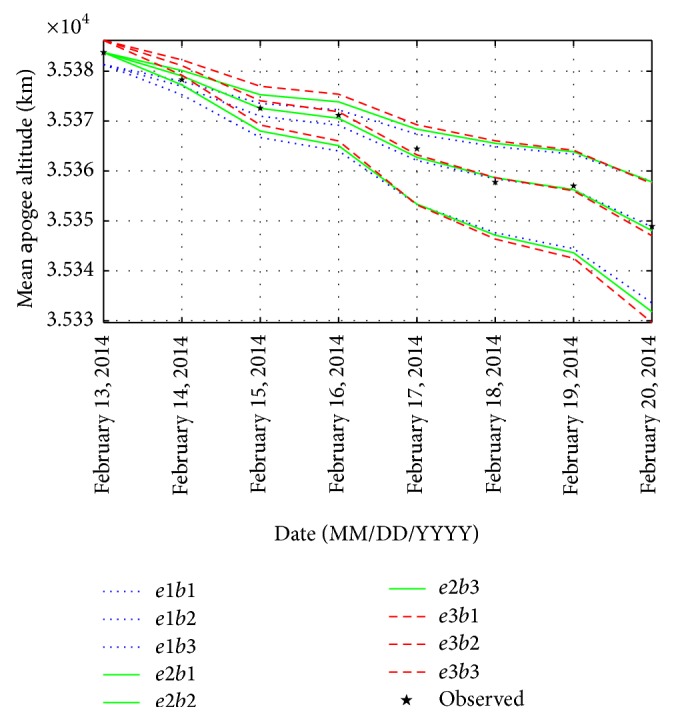
Set of mean apogee surfaces for zone A with (*e*1, *e*2, *e*3) = (0.7278715192, 0.7279715192, 0.7280715192) and (*b*1, *b*2, *b*3) = (60, 80, 100).

To generate a set of mean apogee surfaces for zone A, three values of initial osculating eccentricity (0.7278715192, 0.7279715192, and 0.7280715192) and three values of ballistic coefficient (60, 80, and 100) are selected to obtain nine grid points as plotted in Figure 4. The mean apogee altitudes for the osculating eccentricity *e*1 and the ballistic coefficient *b*1 are predicted using the NPOE software. Similarly, the mean apogee altitudes for (*e*1, *b*2), (*e*1, *b*3), (*e*2, *b*1), (*e*2, *b*2), (*e*2, *b*3), (*e*3, *b*1), (*e*3, *b*2), and (*e*3, *b*3) are predicted using the NPOE software and plotted using MATLAB. Using the genetic algorithm, the values of *e* and *B* are obtained as 0.727963865 and 84.4065933 kg/m^2^, respectively. With the initial estimates of *e* and *B*, the reentry epoch of GSLV-D5 is found to be on June 1, 2014 (147 days from January 5, 2014). The results are given in Table 1.

The number of chromosomes is 24 and the mating pool is

 Mate = $\begin{array}{ccccc} 00101 & 11101 & 01111 & 00011 & 0111 \end{array}$

A number of crossovers and mutations were performed on the chromosomes to get the best solution. The fitness or the quality of the solution is determined by

(2) $$\begin{array}{c} F_{a}=\sqrt{\frac{\sum_{k=1}^{N}{\left(h_{a\text{obs}}\left(t_{k}\right)-\left\{h_{ak}\left(B,e\right)\right\}\right)}^{2}\;}{N}}, \end{array}$$

where *F*_*a*_ is the average dispersions in apogee, *h*_*a*obs_ is observed apogee altitude, *e* is the osculating eccentricity, *t* is time instants for observations, *h*_*ak*_(*B*, *e*) is apogee surface, where *k* = 1,2,…*N*, and *N* is the number of observations.

#### 3.1.2. Zone B

The osculating orbital elements of GSLV R/B as obtained from the TLE on March 2, 2014, 10 : 45 (UTC), are as follows:

 semimajor axis (km) = 24087.380496,
 eccentricity = 0.7279210409,
 inclination (°) = 19.33807553,
 argument of perigee (°) = 219.63775080,
 right ascension of the ascending node (°) = 189.22925587,
 true anomaly (°) = 140.11376942.

**Figure 5:**
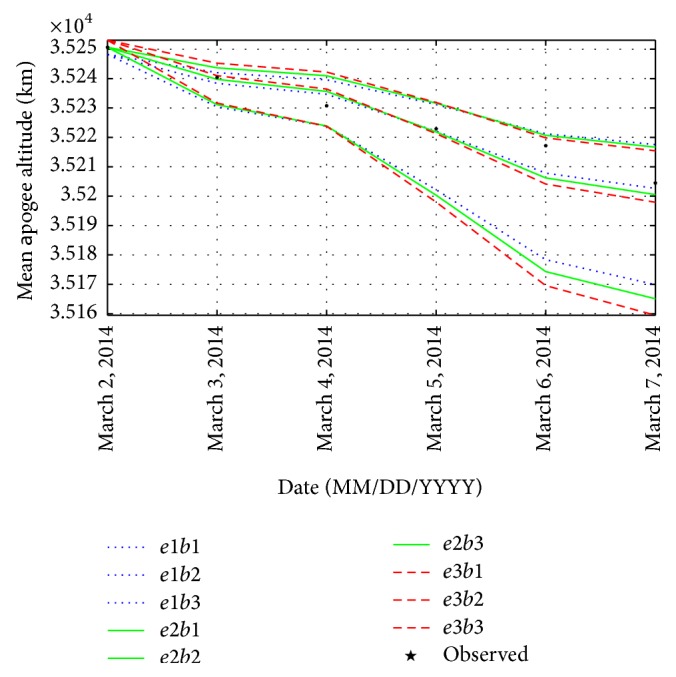
Set of mean apogee surfaces for zone B with (*e*1, *e*2, *e*3) = (0.7278210409, 0.7279210409, 0.7280210409) and (*b*1, *b*2, *b*3) = (50, 80, 110).

To generate a set of mean apogee surfaces for zone B, three values of initial osculating eccentricity (0.7278210409, 0.7279210409, and 0.7280210409) and three values of ballistic coefficient (50, 80, and 110) are selected to obtain nine grid points as plotted in Figure 5. Using the genetic algorithm, the values of *e* and *B* are obtained as 0.727896214 and 85.8965302 kg/m^2^, respectively.

With the initial estimates of *e* and *B*, the reentry epoch of GSLV-D5 is found to be on June 1, 2014 (147 days from January 5, 2014). The results are given in Table 2.

#### 3.1.3. Zone C

The osculating orbital elements of GSLV R/B as obtained from the TLE on March 10, 2014, 14 : 32 (UTC), are as follows:

 semimajor axis (km) = 24031.141029,
 eccentricity = 0.7277057104,
 inclination (°) = 19.33698821,
 argument of perigee (°) = 225.83231865,
 right ascension of the ascending node (°) = 185.82625378,
 true anomaly (°) = 133.79218860.

**Figure 6:**
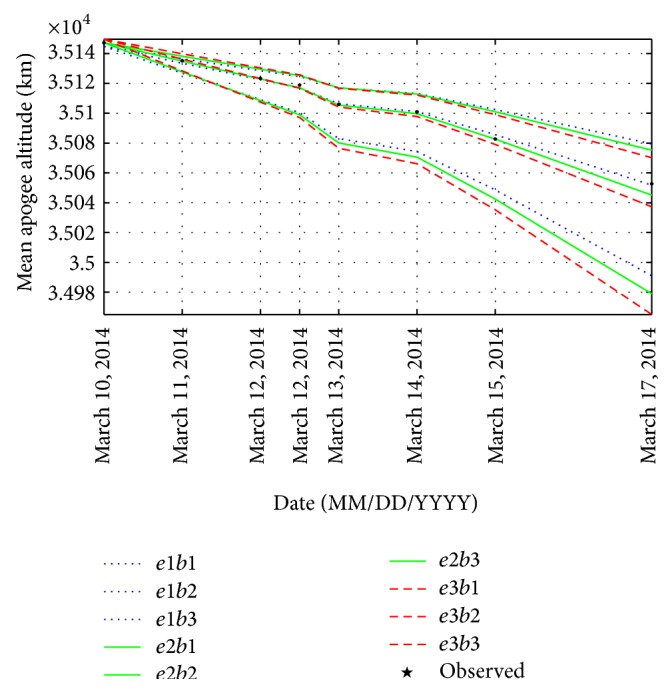
Set of mean apogee surfaces for zone C with (*e*1, *e*2, *e*3) = (0.7276057104, 0.7277057104, 0.7278057104) and (*b*1, *b*2, *b*3) = (50, 80, 110).

To generate a set of mean apogee surfaces for zone C, three values of initial osculating eccentricity (0.7276057104, 0.7277057104, and 0.7278057104) and three values of ballistic coefficient (50, 80, and 110) are selected to obtain nine grid points as plotted in Figure 6. Using the genetic algorithm, the values of *e* and *B* are obtained as 0.727617443 and 81.6301346 kg/m^2^, respectively.

With the initial estimates of *e* and *B*, the reentry epoch of GSLV-D5 is found to be on June 2, 2014 (148 days from January 5, 2014). The results are given in Table 3.

#### 3.1.4. Zone D

**Figure 7:**
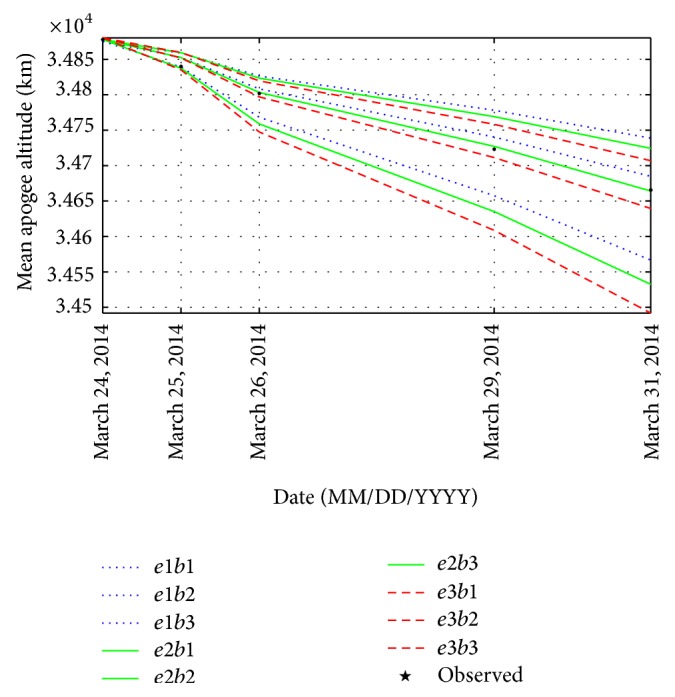
Set of mean apogee surfaces for zone D with (*e*1, *e*2, *e*3) = (0.7267822734, 0.7268822734, 0.7269822734) and (*b*1, *b*2, *b*3) = (50, 80, 110).

The osculating orbital elements of GSLV R/B as obtained from the TLE on March 24, 2014, 06 : 31 (UTC), are as follows:

 semimajor axis (km) = 23888.894645,
 eccentricity = 0.7268822734,
 inclination (°) = 19.29960868,
 argument of perigee (°) = 236.26578322,
 right ascension of the ascending node (°) = 180.05835079,
 true anomaly (°) = 121.02982099,

To generate a set of mean apogee surfaces for zone D, three values of initial osculating eccentricity (0.7267822734, 0.7268822734, and 0.7269822734) and three values of ballistic coefficient (50, 80, and 110) are selected to obtain nine grid points as plotted in Figure 7. Using the genetic algorithm, the values of *e* and *B* are obtained as 0.726806283 and 76.4813202 kg/m^2^, respectively. With the initial estimates of *e* and *B*, the reentry epoch of GSLV-D5 is found to be on May 29, 2014 (144 days from January 5, 2014). The results are given in Table 4.

From the above four zones, the orbital lifetime of GSLV-D5 is found to be between 144 and 148 days, which is a small variation between the four lifetime values from four different epochs. The near linear variation of mean apogee altitude has shown the reentry time more accurately from the TLEs considered. Hence, it is proven once again that the method based on near linear variation of mean apogee altitude utilized in [6] is providing reasonably good estimates of the orbital lifetime of the rocket body. The genetic algorithm parameters are given in Table 5. The computed values of *e* and *B*, the predicted reentry time, and time interval are given in Table 6.

The justification for using RSM to predict mean apogee altitude, as opposed to using NPOE software to propagate the trajectory forward (starting with the initial values of *e* and *B* possessed by each population member in the GA) to the observed epoch time of the TLE, is the computational savings of interpolation over numerical propagation. While the present methodology is not guaranteed to improve reentry estimates in every scenario, these results indicate that the RSM technique combined with a GA is a promising approach. Moreover, the objects from GTO usually reenter after a long time but this object reenters between 144 and 148 days, making this paper very important for space mitigation efforts. The idea of identifying near linear variation of mean apogee altitude time intervals for reentry time prediction has helped to get better results with less time consumption.

## 4. Conclusions 

The response surface technique with the genetic algorithm is utilized to obtain the optimal values of the initial osculating eccentricity and the ballistic coefficient of each of the selected time intervals based on the near linear variation of mean apogee altitude. Using these optimal values, the orbital lifetime of a GSLV-D5 rocket body is found to be between 144 and 148 days from its injection into the orbit on January 5, 2014.

## Figures and Tables

**Table 1:** Observed and predicted mean apogee altitude for zone A.

TLE epoch (UTC)	Mean apogee observed (km)	Mean apogee predicted (km)	Error (km)
February 13, 2014, 15:24	35383.75000	35383.56640	0.184
February 14, 2014, 12:08	35378.30469	35379.12109	−0.816
February 15, 2014, 19:12	35372.59375	35373.03125	−0.438
February 16, 2014, 05:33	35371.17969	35371.17578	0.004
February 17, 2014, 22:59	35364.45313	35363.92578	0.527
February 18, 2014, 19:41	35357.75781	35360.13672	−2.379
February 19, 2014, 06:02	35357.01172	35357.91797	−0.906
February 20, 2014, 13:05	35348.90234	35350.23828	−1.336

**Table 2:** Observed and predicted mean apogee altitude for zone B.

TLE epoch (UTC)	Mean apogee observed (km)	Mean apogee predicted (km)	Error (km)
March 2, 2014, 10:45	35250.6328125	35250.03125	0.602
March 3, 2014, 17:43	35240.3906300	35240.16016	0.230
March 4, 2014, 04:01	35230.7539100	35236.41406	−5.660
March 5, 2014, 10:58	35222.8984400	35223.78516	−0.887
March 6, 2014, 17:53	35217.1718800	35209.39844	7.773
March 7, 2014, 04:11	35204.4765600	35204.16797	0.309

**Table 3:** Observed and predicted mean apogee altitude for zone C.

TLE epoch (UTC)	Mean apogee observed (km)	Mean apogee predicted (km)	Error (km)
March 10, 2014, 14:32	35147.26563	35145.14063	2.125
March 11, 2014, 11:07	35135.21484	35133.85938	1.355
March 12, 2014, 07:41	35123.41797	35122.85547	0.563
March 12, 2014, 17:57	35118.95313	35117.47266	1.480
March 13, 2014, 14:31	35105.96094	35106.90625	−0.945
March 14, 2014, 00:47	35100.91406	35101.61328	−0.699
March 15, 2014, 07:35	35082.73828	35086.21484	−3.477
March 17, 2014, 21:07	35052.68750	35052.47656	0.211

**Table 4:** Observed and predicted mean apogee altitude for zone D.

TLE epoch (UTC)	Mean apogee observed (km)	Mean apogee predicted (km)	Error (km)
March 24, 2014, 06:31	34877.94531	34876.13281	1.813
March 25, 2014, 02:58	34839.96094	34850.94141	−10.980
March 26, 2014, 19:43	34801.82031	34802.17969	−0.359
March 29, 2014, 08:41	34723.11328	34727.16016	−4.047
March 31, 2014, 01:15	34665.50781	34665.56250	−0.055

**Table 5:** Parameters of the genetic algorithm.

Zone label	Number of chromosomes	Number of GA generations to converge	Crossover probability	Mutation probability
A	24	15	0.8	0.01
B	32	9	0.8	0.01
C	36	23	0.8	0.01
D	34	18	0.8	0.01

**Table 6:** Computed values of initial osculating eccentricity and ballistic coefficient and reentry time for each zone using RSM with GA.

Zone label	TLEs considered (UTC)		Computed values		Predicted reentry time (mm-dd-yyyy)	Time interval^a^ (days)
	From	To	Initial osculating eccentricity (*e*)	Initial ballistic coefficient (*B*)		
A	February 13, 2014, 15:24	February 20, 2014, 13:05	0.72796	84.4065933	June 1, 2014	147
B	March 2, 2014, 10:45	March 7, 2014, 04:11	0.72789621	85.8965302	June 1, 2014	147
C	March 10, 2014, 14:32	March 17, 2014, 21:07	0.72761744	81.6301346	June 2, 2014	148
D	March 24, 2014, 06:31	March 31, 2014, 01:15	0.72680628	76.4813202	May 29, 2014	144

^a^Time interval is the number of days between the predicted reentry time and the first TLE epoch of zone A.

## Nomenclature

GTO: Geostationary transfer orbit
GSLV: Geosynchronous satellite launch vehicle
LEO: Low Earth orbit
GSO: Geostationary orbit
*C*_*D*_: Drag coefficient
*m*: Mass of the object
*A*: Effective area
R/B: Rocket body
TLE: Two-line element
GA: Genetic algorithm
NPOE: Numerical prediction of orbital events
SGP: Simplified general perturbations
SDP: Simplified deep space perturbations
*F*_10.7_: Solar flux
*A*_*p*_: Geomagnetic index
RSM: Response surface methodology
*x*_1_, *x*_2_: Independent variables
*ɛ*: Random error
*y*: Response
EA: Evolutionary algorithms
*e*: Osculating eccentricity
*B*: Ballistic coefficient
A, B, C, D: Four zones
*J*_10,10_: Zonal and tesseral harmonic terms
GEM10B: Goddard Earth model 10B
MSIS90: Mass-Spectrometer-Incoherent-Scatter-1990 atmosphere model
NORAD: North American Aerospace Defense Command
UTC: Coordinated universal time
(*e*1, *e*2, *e*3): Three values of osculating eccentricity
(*b*1, *b*2, *b*3): Three values of ballistic coefficient
*e*1*b*1: First value of osculating eccentricity and ballistic coefficient
*e*1*b*2: First value of osculating eccentricity and second value of ballistic coefficient
*F*_*a*_: Average dispersions in apogee
*h*_*a*obs_: Observed apogee altitude
*h*_*ak*_(*B*, *e*): Apogee surface
*t*: Time instants for observations
*N*: Number of observations
*k*: 1,2,…*N*.
